# Colostomy using a percutaneous lumen-apposing metal stent

**DOI:** 10.1055/a-2173-7520

**Published:** 2023-10-24

**Authors:** Kirill Basiliya, Jurjen J. Boonstra, Akin Inderson

**Affiliations:** Department of Gastroenterology and Hepatology, Leiden University Medical Center, Leiden, The Netherlands

A 55-year-old man with a history of Crohn’s disease with multiple abdominal resections, poor wound healing, and rectal cancer with pulmonary metastasis presented with abdominal distension, pain, and absence of bowel movement. A computed tomography scan showed a distended colon, with the known obstructing malignant lesion in the rectum. A surgical colostomy was judged not to be possible owing to his history of multiple and extensive abdominal surgical procedures. A colonic stent was not possible owing to the distal position and length of the lesion.

After discussion with the patient and inspired by the case report of Canakis and Baron 1 , we decided to place a percutaneous lumen-apposing metal stent to act as a colostomy ( Video 1 ). A pediatric gastroscope could be passed alongside the malignant stricture. The appropriate position of the scope was confirmed using indentation and transillumination. The large bowel was sutured to the abdominal wall using the Freka Pexact gastropexy device (Fresenius Kabi). A Chiba needle was used to introduce a guidewire, over which a 16 × 10-mm Niti-S Hot SPAXUS stent (Taewoong Medical) was successfully deployed ( Fig. 1 ).

**Video 1** A colostomy is created using a percutaneous lumen-apposing metal stent.


**Fig. 1:**
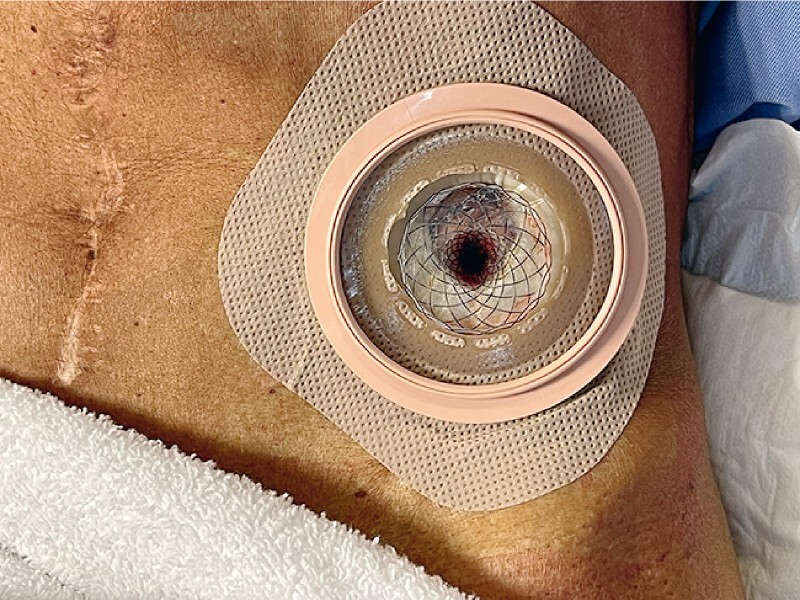
Photograph of a percutaneous colostomy created using a lumen-apposing metal stent.

Over the following days, the stent did not deploy sufficiently and attempted CRE balloon dilation up to 15 mm was unsuccessful. A decision was made to incise the abdominal fascia around the stent, after which it deployed fully and functioned as a colostomy for at least the next 3 weeks, at which time the patient was discharged home with palliative care.

Endoscopy_UCTN_Code_TTT_1AQ_2AF

## Notes

Endoscopy E-Videos

https://eref.thieme.de/e-videos
*E-Videos* is an open access online section of the journal *Endoscopy* , reporting on interesting cases and new techniques in gastroenterological endoscopy. All papers include a high-quality video and are published with a Creative Commons CC-BY license. Endoscopy E-Videos qualify for HINARI discounts and waivers and eligibility is automatically checked during the submission process. We grant 100% waivers to articles whose corresponding authors are based in Group A countries and 50% waivers to those who are based in Group B countries as classified by Research4Life (see: https://www.research4life.org/access/eligibility/ ). This section has its own submission website at https://mc.manuscriptcentral.com/e-videos
